# Euglycemic Diabetic Ketoacidosis and Sodium-Glucose Cotransporter-2 Inhibitors: A Focused Review of Pathophysiology, Risk Factors, and Triggers

**DOI:** 10.7759/cureus.13665

**Published:** 2021-03-03

**Authors:** Manoj R Somagutta, Kuchalambal Agadi, Namrata Hange, Molly S Jain, Erkan Batti, Bernard O Emuze, Elizabeth O Amos-Arowoshegbe, Sorin Popescu, Saad Hanan, Varadha Retna Kumar, Kezia Pormento

**Affiliations:** 1 Department of Medicine, California Institute of Behavioral Neurosciences & Psychology, Fairfield, USA; 2 Department of Medicine, Avalon University School of Medicine, Willemstad, CUW; 3 Clinical Research, Larkin Health System, Chicago, USA; 4 Public Health, Woodlands Health Campus, Singapore, SGP; 5 Department of Medicine, Saint James School of Medicine, Park Ridge, USA; 6 Department of Medicine, Washington University Health and Science, San Pedro, BLZ; 7 Emergency Medicine, Saint James School of Medicine, Fort Worth, USA; 8 Department of Medicine, Windsor University School of Medicine, Basseterre, KNA; 9 Department of Nephrology, Lourdes Hospital, Kochi, IND; 10 Department of Medicine, Ateneo de Manila School of Medicine and Public Health, Quezon City, PHL

**Keywords:** euglycemic diabetic ketoacidosis, edka, canagliflozin, empagliflozin, diabetes, sodium, risk factors, diabetic ketoacidosis, sodium-glucose cotransporter 2 inhibitor

## Abstract

Diabetic ketoacidosis (DKA) is an acute and significant life-threatening complication of diabetes. The association of sodium-glucose cotransporter-2 inhibitors (SGLT2i) with euglycemic diabetic ketoacidosis (EDKA) has been well reported. This literature review was conducted to understand the mechanism of EDKA and identify the potential risk factors and precipitants for patients taking SGLT2i. After reviewing the published literature between 2010 and 2020, 32 articles are included in the final review. The underlying mechanism is mainly enhanced lipolysis and ketone body reabsorption. SGLT2i also stimulates pancreatic alpha cells and inhibits beta cells, causing an imbalance in glucagon/insulin levels, further contributing to lipolysis and ketogenesis. Most patients were diagnosed with blood glucose less than 200 mg/dL, blood pH <7.3, increased anion gap, increased blood, or urine ketones. Perioperative fasting, pancreatic etiology, low carbohydrate or ketogenic diet, obesity, and malignancy are identified precipitants in this review. As normoglycemia can conceal the underlying acidosis, physicians should be cognizant of the EDKA diagnosis and initiate prompt treatment. Patient education on risk factors and triggers is recommended to avoid future events.

## Introduction and background

Diabetic ketoacidosis (DKA) is a common, life-threatening complication seen in patients with type 1 and type 2 diabetes mellitus (T2DM) [1]. The following three main findings mark the diagnosis: high anion gap metabolic acidosis (pH <7.3 and serum bicarbonate <15 mEq/dL), ketone bodies in the blood and/or urine, and high blood sugar levels ranging 250-600 mg/dL [2]. In a subgroup of patients with diabetes, ketonemia and metabolic acidosis occur without a concurrent rise in blood sugar levels (<200 mg/dL), which poses a diagnostic challenge for euglycemic diabetic ketoacidosis (EDKA). Munro et al. first described this occurrence in 1973 in a study involving 211 patients with DKA, of which 37 exhibited a normal glycemic status with ketoacidosis. With the emergence of oral hypoglycemic agents like sodium-glucose cotransporter inhibitors (SGLT2i), EDKA has been reportedly rising [3].

The increased prevalence of EDKA is seen with SGLT2i, which are federally approved medications to treat T2DM [4]. The exceptionally high incidence of T2DM across different ethnic groups has made it extremely important for physicians to understand the complications of the drugs used to treat this disease. However, there is mounting evidence of additional causes of EDKA. The common hypothesis in all factors triggering euglycemia is a state of significant carbohydrate deficit that leads to hypoinsulinemia [5,6]. Insulinopenia, along with an excess of counterregulatory hormone production, results in an increased glucagon insulin ratio, which accelerates lipolysis and ketonemia [6,7]. The counterregulatory hormone production is especially significant in stress on the body, such as surgery or sepsis [2,7].

Limited studies have discussed EDKA; however, its increasing incidence warrants a more thorough investigation to prompt physicians worldwide to consider ketosis in a diabetic patient despite their normal serum glucose levels. In a meta-analysis published by Burke et al. in 2017, the leading risk factors that predispose an individual on SGLT2i to DKA include medication noncompliance, infection, major surgeries, and underlying autoimmune diabetes in patients previously diagnosed with T2DM [7]. Other precipitating factors for euglycemic EDKA are similar to hyperglycemic DKA and include starvation, infarction, pregnancy, ketogenic diet, low carbohydrate diet, dehydration, recent reduction in insulin dose, and extreme physical activity, among others [2-10]. This review summarizes the various risk factors, triggering agents, pathophysiology, and therapeutic methods involved.

## Review

Methods and results

**Figure 1:**
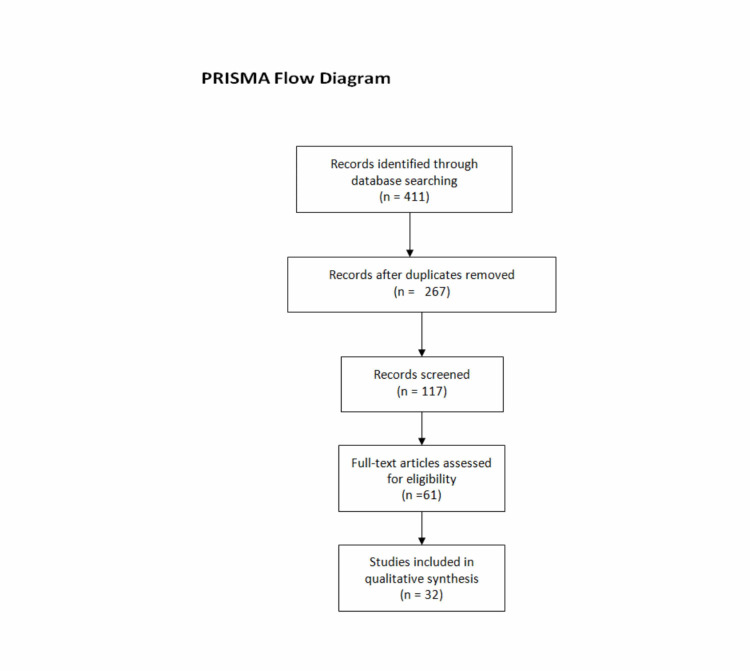
Flow chart explaining the process of the literature review.

A specific literature search was performed to identify articles for EDKA risk factors and precipitants for patients on SGLT2i. We searched numerous databases such as PubMed, Google Scholar, Web of Science, and Scopus to search for published literature on this topic published between 2010 and 2020. A detailed literature search of the articles referenced in the identified publications was also performed. The keywords used included but are not limited to “euglycemic diabetic ketoacidosis,” “Sodium-glucose cotransporter inhibitors,” “EDKA risk factors,” “EDKA in dapagliflozin,” “EDKA and canagliflozin,” “EDKA and empagliflozin,” “EDKA and SGLT2i.” The inclusion criteria included case reports published between 2010 and 2020, as well as articles available in the English language. The exclusion criteria consisted of duplicate articles or abstracts only, animal studies, studies published before 2010, and articles published in languages other than English. The initial screening of abstracts yielded 411 articles. We carefully reviewed these 411 abstracts and removed duplicates, those that did not meet our inclusion criteria, and those for which full articles were not available for free. Upon screening, we finalized 33 articles to be included in this review. The PRISMA flowchart represents the article screening process to the final included articles (Figure 1).

Discussion

Sodium-Glucose Cotransporter 2

Sodium-glucose cotransporter (SGLT2) is a transmembrane protein and, as its name suggests, cotransports sodium and glucose across the cell membrane. Although there are six isoforms, two important forms are type 1 and 2, with the former located on the intestinal cells, and the latter mostly on the apical cells of the proximal convoluted tubules in the kidney and alpha cells of the pancreas [10,11]. These transporters are driven by the electrochemical gradient generated by the basolateral sodium/potassium-ATPase pump, and SGLT2 aids in the reabsorption of glucose from the proximal tubule segment 1 and 2 [11-13]. In 2013-2014, the Food and Drug Administration (FDA) approved the clinical use of SGLT2i such as canagliflozin, dapagliflozin, and empagliflozin to treat T2DM, either as monotherapy or in combination with other antidiabetic drugs. Its action mechanism leading to a decreased dose of insulin required in T1DM patients made it an off-label drug to manage T1DM [10]. Different clinical trials have well established the cardio-renal protective effect of SGLT2i [12]. For instance, the EMPA-REG OUTCOME trial was conducted to assess the effect of SGLT2i use in T2DM patients with a high cardiac risk. The researchers observed relative risk reductions in cardiovascular (CV) mortality by 38%, hospitalization for heart failure by 35%, and death from any cause by 32% [13]. The researchers hypothesize that: (i) in mild hyperketonemia caused by SGLT2i, beta-hydroxybutyrate is freely taken up by the cells as a source of energy, especially by the cardiac muscles. Use of this energy source helps the tissues work efficiently. (ii) Glucosuria and ketonuria cause a mild hemoconcentration ensuring more efficient delivery of oxygen to the tissues. Since then, the American Diabetic Association (ADA) recommends its inclusion in treating T2DM with nephropathy and/or congestive heart failure [14]. The known side effects of SGLT2i were urinary tract infection (especially with canagliflozin), osmotic diuresis leading to increased urination, thirst, dyslipidemia, and increased excretion of uric acid. Nappi et al. reported a case of EDKA in a patient taking SGLT2i (empagliflozin) along with metformin [15]. In this patient, they noticed a concurrent acute kidney injury due to the severe osmotic diuresis apart from the delayed diagnosis of EDKA. Canagliflozin was also associated with increased amputation. In the post-marketing surveillance, it was found that SGLT2i caused EDKA. In 2015, FDA reported 73 cases of EDKA with the use of SGLT2i [16,17]. Some researchers reason that because EDKA can occur even in those not taking SGLT2i, the true incidence of EDKA in those taking SGLT2i is not known.

Pathophysiology

**Figure 2:**
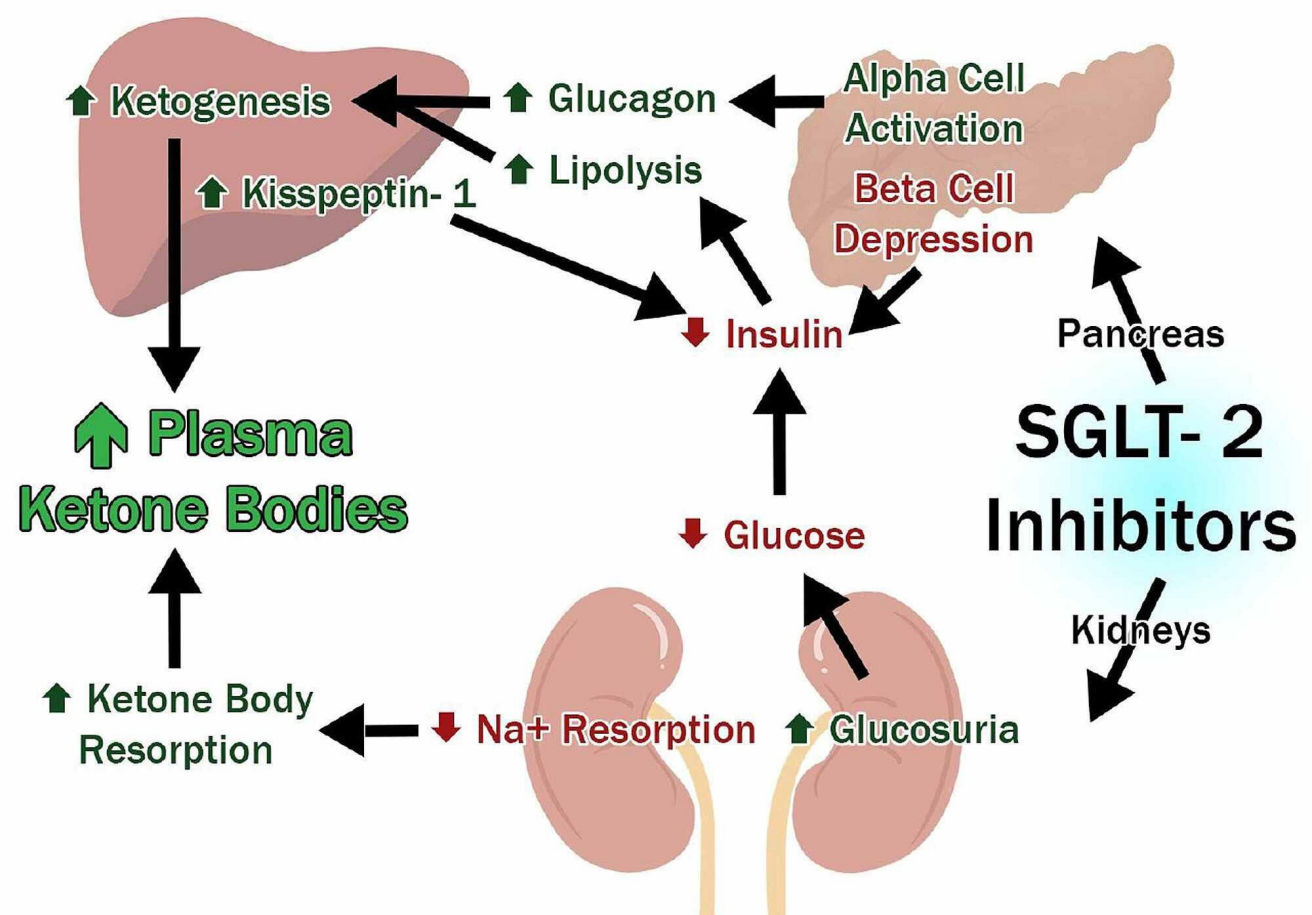
Pathophysiology and mechanism of SGLT2i causing EDKA. SGLT2 = sodium-glucose cotransporter-2, Na^+^ = sodium; EDKA = euglycemic diabetic ketoacidosis

The underlying mechanism of SGLT2i causing EDKA is multifactorial and shown in Figure 2 [16-19].

SGLT2i on kidney enhance glucose excretion and causes the reabsorption of ketone bodies. In pancreas, there is contemplation that SGLT2i inhibit beta cells causing increased lipolysis, adding to the pool of ketone bodies [16-18]. They also have been found to stimulate alpha cells of the pancreas, inciting the release of glucagon and causing an imbalance in glucagon/insulin levels [18,19]. The studies so far have shown that EDKA in patients taking SGLT2i is more common among patients who have had diabetes for a long time; and there are other factors precipitating it. We reviewed the included articles for the risk factors and precipitants for EDKA in patients taking SGLT2i. We noticed 11 cases associated with pre/postoperative fasting, three associated with pancreatic etiology, five associated with low carbohydrate or keto diet, one with malignancy, and one in an obese adolescent [20-33].

**Figure 3:**
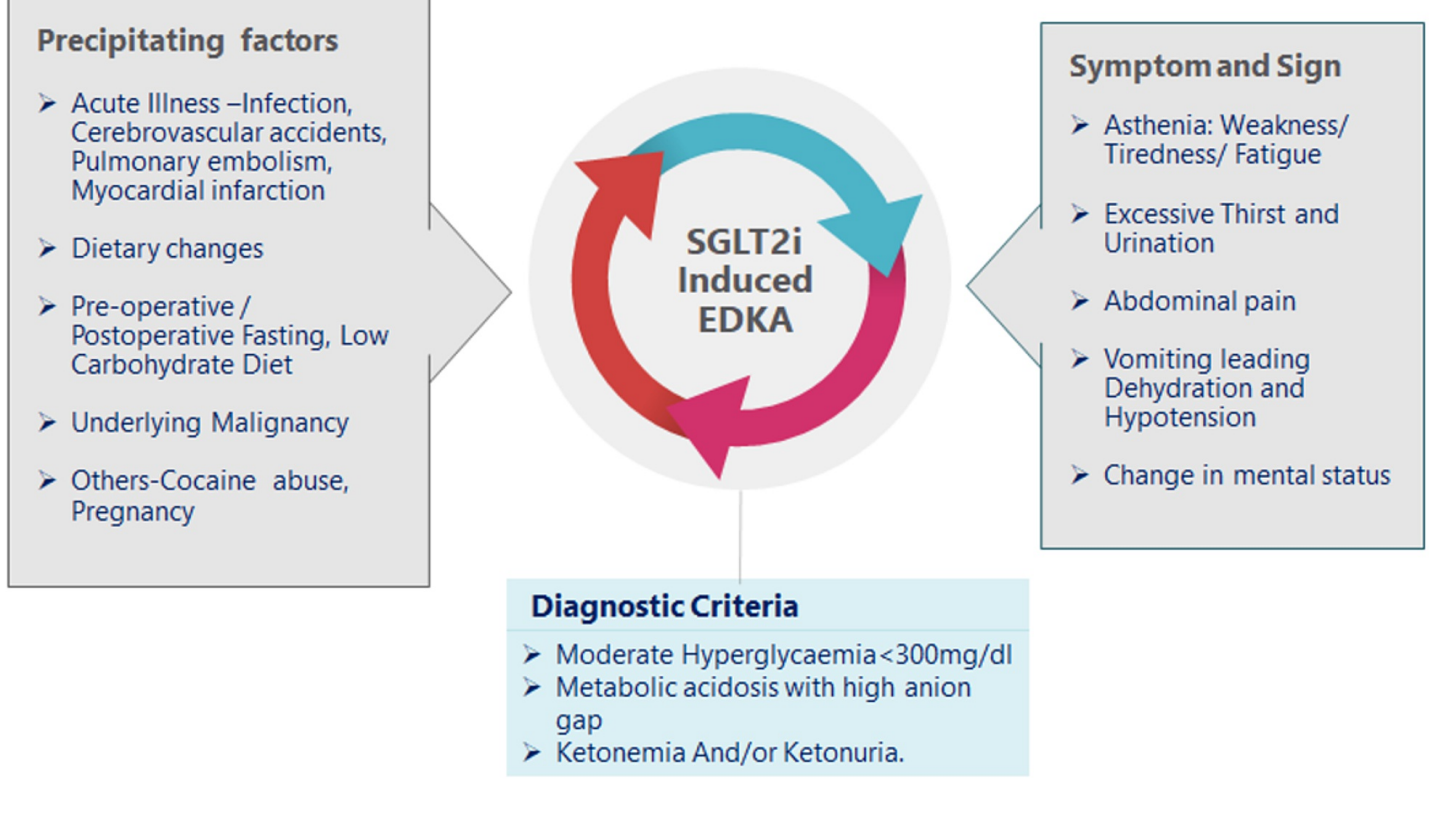
Precipitating factors, symptoms, and diagnostic criteria for SGLT2i-associated EDKA. SGLT2i = sodium-glucose cotransporter-2 inhibitors; EDKA = euglycemic diabetic ketoacidosis

The majority of the authors reported EDKA in middle-aged women with long-standing T2DM, body mass index between 35 and 40 kg/m^2^, and who had been on multiple doses of SGLT2i for at least more than three months [34-36]. The common presenting complaints were fatigue, abdominal pain, shortness of breath, reduced food intake, nausea, and vomiting. On examination, they were tachypneic, with tachycardia, blood glucose less than 200 mg/dL, blood pH <7.3 (7.08-7.2), increased anion gap, and increased blood ketones, such as beta-hydroxybutyrate and serum acetone. Sampani et al. reported increased ketones in the urine as well [34]. The precipitating factors, signs and symptoms, and diagnostic criteria are depicted in Figure 3 [2,7-10,20-32,34].

Precipitating factors

Dietary Factors

Since the 1960s, ketogenic or very low carbohydrate diets (VLCDs) have been widely recommended for weight loss. The popularity of this diet has increased with many studies proving its beneficial effects in treating patients with polydrug therapy for a seizure disorder, slowing the progression in Alzheimer’s disease, improving the glucose tolerance in insulin-resistant DM, weight loss, and other neurodegenerative diseases [37]. The science behind this diet is to forcefully induce “ketosis” in individuals by restricting the carbohydrate intake to less than 50 g daily over several days. In some patients with diabetes, prescribing SGLT2i (which is also known to cause ketosis) or unsupervised keto diet initiation in patients who are already taking SGLT2i has placed them at risk of life-threatening EDKA [29].

SGLT2i causes the loss of glucose in the urine and increases the glucagon/insulin ratio resulting in increased gluconeogenesis, glycogenolysis, and fatty acid metabolism leading to weight loss [30]. VLCD has been proven to promote weight loss and is frequently used in the dietary management of T2DM [31]. Few authors have recorded case reports of EDKA precipitated by conditions where both SGLT2i and VLCD were instituted together [30,38]. Both the women adhered to a diet with less than 20 g of carbohydrate, with reported weight loss of more than 5 pounds in a month. They presented to the hospital with tachypnea, tachycardia, and fatigue, with blood pH of <7.2 and elevated blood ketones. They were treated successfully in the intensive care setting, with large boluses of fluids, electrolyte correction, and insulin. In both cases, physicians restarted the low carbohydrate dietary management with very close monitoring. They started carbohydrate intake of about 75 g a day and gradually reduced it. Shaika et al. reported a case of EDKA in a T1DM patient who had started a keto diet [31]. This case illustrates the importance of educating diabetic patients about the need to consult with their primary care provider before commencing a dietary or exercise change. The authors recommended further studies to analyze the long-term risk versus benefit of a low carbohydrate diet in diabetic patients as it causes weight loss and alters lipids by increasing low-density lipoproteins and triglycerides [31].

Hypertriglyceridemia

Two of the articles we reviewed reported cases of EDKA in the setting of SGLT2i use in patients with preexisting hypertriglyceridemia. Badwal et al. reported a case of EDKA in a 25-year-old male with a history of pancreatitis secondary to hypertriglyceridemia and diabetes managed with metformin, sitagliptin, and dapagliflozin [37]. Gajjar et al. reported the case of a 28-year-old women on dapagliflozin and metformin for T2DM management and with triglyceride level of more than 500 mg/dL [39].

In both cases, patients presented with abdominal pain, nausea, and vomiting. Their initial labs showed normal glucose, increased anion gap, increased HbA1c levels, and hypertriglyceridemia of >5,000 mg/dL and >2,000 mg/dL, respectively. In both cases, the treating attendees diagnosed EDKA with elevated beta-hydroxybutyrate levels, and treated the patients with intensive fluid replacement, insulin infusion (discontinued all oral hypoglycemic agents), bicarbonates, and electrolytes. The former was also diagnosed with pancreatitis considering elevated lipase, while in the latter, the lipase was found to be within normal limits. Both patients reported a complete recovery and were discharged on insulin. The authors emphasized that inadequate oral intake is a confounding factor leading to ketosis and EDKA in their patients. They also highlight the importance of ordering beta-hydroxybutyrate levels to clinch the diagnosis of EDKA in suspected cases and restarting SGLT2i only after consultation with the endocrinologist.

Perioperative Fasting

The half-life of a single dose of SGLT2i varies between eight and 16 hours. Patients on a multiple-dose regimen for a prolonged time have a longer half-life [36]. In perioperative situations, patients are in a fasting state, and if the patients are on SGLT2i, they continue to lose glucose in the urine, more ketones will be reabsorbed into the tubules, and are synthesized in the liver by lipolysis. These collectively result in ketonemia and an acidotic state. These patients may also have multiple factors that might cause the acidotic state, such as starvation and possible underlying or complicating infection, and clinicians should be cautious about ruling out DKA, especially the euglycemic variety. These cases are tabulated in Table 1.

**Table 1:** Cases of EDKA in patients receiving SGLT2i triggered by pre/postoperative fasting. Yrs = years; F = female; M = Male; PMH = past medical history; T2DM = type 2 diabetes mellitus; SOB = shortness of breath; HTN = hypertension; OSA: obstructive sleep apnea; AMS = altered mental status; CRRT = continuous renal replacement therapy; AOCKI IV = acute on chronic stage IV kidney injury; Cx = complicated; POD = postoperative day; ABG = arterial blood gas; AG = anion gap; BG = blood glucose; BUN = blood urea nitrogen; EDKA = euglycemic diabetic ketoacidosis

Author	Clinical profile	Signs & symptoms	Time of event	Lab values	Treatment
Banakh et al., 2019 [20]	64 Yrs, F with T2DM, HTN, hypercholesterolemia	Dehydration, tachypnoea, tachycardia	POD-4 gastric sleeve surgery	Glu = 13.5 mmol/L, ABG: pH = 6.93, HCO_3_ = 2 mmol/L, K^+^ = 4.3 mmol/L, blood ketones = elevated, lactate = 1.5 mmol/L	ED: rapid rehydration with 3 L of NS with 10% dextrose and NS >300 mL of 8.4% NaHCO_3_
Segebrecht et al., 2019 [21]	65 Yrs, F with T2DM, CKD stage IV	AMS, tachypnea, gastrointestinal bleeding, diarrhea	POD-11 abdominal surgery for incarcerated hernia	BG = 118 mg/dL, pH = 7.08, HCO_3_ = 14, beta-hydroxybutyrate (ketones) = 8.9 mmol/L AG = 12, lactate = 0.4 mmol/L, BUN = 10, SCr = 0.7	CRRT for AOCI, IV fluid replacement with insulin drip, 50% D
Segebrecht et al., 2019 [21]	75 Yrs, M with T2DM, AOCKD: RT done	AMS	POD-0 exploratory laparotomy with right hemicolectomy and ileocolic anastomosis	AG = 18, HCO_3_ = 12, Na = 141, beta-hydroxybutyrate = 5.0 mmol/L, lactate = 1.5 mmol/L	IV fluid, insulin, electrolytes
Jaberi et al., 2016[22]	47 Yrs, F with T2DM, Graves disease, post-cholecystectomy, hyperlipidemia, depression	AMS nausea, vomiting, dehydration	POD-2 after hip replacement surgery	BG = 152 mg/dL, pH = 7.18 K^+^ = 4.4 mEq/L AG = 17, HCO_3_ = 9.2 mg/dL, ketonuria = 2+, HCO_3_ = 17, lactate = 1 mmol/L	IV fluid, insulin, electrolytes
Mackintosh et al., 2020 [23]	68 Yrs, F with T2DM	Increasingly lethargic with confusion and worsening expressive aphasia	POD-1 craniotomy with tumor excision	BG = 160 mmol/L, HCO_3_ = 9 mmol/L, AG = 21, pH = 7.2 beta-hydroxybutyrate = high, ketonuria ++, glucosuria ++	IV fluid, insulin, electrolytes
Jemma Dowset et al., 2019[24]	43 Yrs, F with T1DM, obesity	Dyspepsia and nausea, decreased oral intake, vomiting	POD-10, post-sleeve gastrectomy	BG = 11.1 mmol/L, ketone = 3.9, pH = 7.26, pCO_2_ = 31 mmHg, HCO_3_ = 13, beta-hydroxybutyrate = 7.4 mmol/L	IV fluid, insulin, electrolytes
Bteich et al., 2019[25]	58 Yrs, F with HTN and OSA	AMS	POD-1, VP shunt with malfunction	BG = 150, pH = 7.20, AG = >28, beta-hydroxybutyrate = 10.09, lactic acid =1.1 mmol/L	IV fluid, insulin, electrolytes
Pace et al., 2018 [26]	66 Yrs, F with T2DM, pancreatic adenocarcinoma, ovarian mass	Polyuria	POD-0, distal pancreatectomy with en bloc splenectomy	BG = WNL HCO_3_ = low, AG = high, beta-hydroxybutyrate = 48.1 mg/dL, Ketonuria = +++	IV fluid, insulin, electrolytes
Pace et al., 2018[26]	75 Yrs, M with T2DM, metastatic cancer	Intraoperative and postoperative polyuria urine output = 100-325 mL/h	POD-0, pylorus-preserving resection pancreas	HCO_3_ = low, AG = 19 mmol/L, beta-hydroxybutyrate = 50.8 mg/dL, lactic acidosis = negative	Fluids with insulin drip was given, frequent lab tests were done
Mohammed Faraz et al., 2019[27]	44 Yrs, F with T2DM, dyslipidemia, obesity	Generalized weakness nausea and anorexia, tachypnea, tachycardia, dehydration	POD-1, post C5-C7 cervical decompression	BG = 9.4 mmol/L, pH = 7.27, HCO_3_ = 18.2 mmol/L, AG = 33.8 ketonuria = 4+, glycosuria = 2+, lactate = 0.8 mmol/L	IV fluid, insulin, electrolytes
Mohammed Faraz et al., 2019 [27]	59 Yrs, F with PMH, T2DM, HTN, PCOS, depression	Generalized weakness, dyspnea, tachypnea, tachycardia, dehydration	POD-3, laparoscopic right partial nephrectomy	Glucose = 12.3 mmol/L, pH = 7.23, HCO_3_ = 9.3 AG = 32.6 ketonuria = 3+, glycosuria = 2+, lactate = 0.5 mmol/L	IV fluid, insulin, electrolytes

One of the advantages of SGLT2i treatment is weight loss. With rapid weight loss, increased lipolysis in the liver results in ketogenesis and precipitates starvation ketosis. In a situation that warrants surgery, the patient is placed on nil per oral, which further aggravates the condition. Banakh et al. reported how EDKA is mistaken for starvation ketosis [20]. Starvation ketoacidosis is usually associated with milder pH of >7.3, serum bicarbonate >18, free fatty acids in serum, and keto-anion levels lower than in EDKA. Most authors have reported altered mental status as a presenting symptom in postoperative patients who were later diagnosed as having EDKA. Pace et al. noted immediate postoperative polyuria [26]. Similar to the management of regular diabetic ketoacidosis, the euglycemic variant is an emergency that needs to be addressed in the intensive care unit with intensive fluid replacement, electrolyte correction, insulin drip, frequent blood draws, and, importantly, stopping the SGLT2i (if it was not done yet). Most authors reported that their patients recovered with the above management. Wang et al. noted that their patient scheduled for revascularization procedure for moyamoya disease suffered from an acute left cerebral infarct and succumbed to the stroke [35]. Segebrecht et al. reported that the antibiotics-induced pseudomembranous colitis precipitated the EDKA in their patient who underwent abdominal surgery [21].

We found that most patients were on SGLT2i therapy for more than six months. Handelsman et al. suggested that SGLT2i drugs be stopped for at least 24-72 hours before the surgery as both stress and surgery can aggravate the risk of ketosis [40]. However, Pace et al. suggested that because SGLT2i have long half-lives, they should be withheld for at least five days before surgery to avoid perioperative ketosis [26]. Even after prompt discontinuation of these drugs, EDKA should be highly suspected and appropriate measures should be taken if these patients present postoperatively with altered mental status, polyuria, glucosuria, tachypnea, tachycardia, or ketosis. They also note that T2DM patients are usually on a multidrug antidiabetic regimen and recommend that physicians’ be vigilant about the rare but noted side effects of hypoglycemic agents, such as pancreatitis due to glucagon-like peptide-1 receptor agonist and heart failure due to di peptidase-4 inhibitors. Bteich et al. added that, in addition to stopping the SGLT-2i preoperatively, these patients should also be started prophylactically on low doses of long-acting insulin [25]. This is because EDKA is precipitated by an imbalance between glucagon/insulin, with the added effect of increased catecholamines and cortisol levels in patients taking SGLT2i [32].

Infection

Muppidi et al. reported a young pregnant woman with T1DM presenting with urinary tract infection symptoms, causing nausea and vomiting in her third trimester of pregnancy [41]. Her blood glucose was <200 mg/dL with ketosis and metabolic acidosis. They treated her aggressively and promptly with fluids, insulin, electrolyte replacement, urinary tract infection treatment, fetal monitoring, and emergency caesarian section. They explained that pregnant women are at an increased risk of acidosis due to factors such as decreased calorie consumption, hormonal changes in the late stages of pregnancy (such as increased estrogen, progesterone, HPL, TNF-alpha, decreased insulin), and respiratory changes in the late stages of pregnancy. Infection, sepsis, and T1DM are also independent factors known to precipitate acidosis. The presence of all factors at once precipitated the severe ketoacidosis state in this case. The usual DKA in a pregnant woman can cause many deleterious effects on maternal and fetal health, including fetal complications due to uterine hypoperfusion, fetal hypoxia, and impaired fetal brain development. The euglycemic state further increases the challenge in diagnosing the condition and treating promptly.

Malignancy

Lucero et al. report a case of EDKA in a 50-year-old woman with hypertension, T2DM, hypothyroidism, dyslipidemia, and left breast cancer, which required treatment with surgery chemoradiation [42]. The patient was treated with 10 mg/day of dapagliflozin. Her labs were pH of 7.13, anionic gap 32, and blood glucose 165 mg/dL, with positive ketonemia. After the diagnosis is made, treatment with a continuous insulin infusion pump was started and the patient was discharged from the hospital after five days. Papadokostaki and Liberopoulos reported the case of a 64-year-old male with T2DM, hypertension presenting with nausea, vomiting, and abdominal pain [32]. He was started on dapagliflozin eight months before his presentation. On further assessment, he had tachycardia and tenderness in the epigastrium and left upper quadrant. In his labs, pH was 7.33, low bicarbonate, K^+^ 4.6 mmol/L, increased anion gap (29 mmol/L), and plasma glucose of 203 mg/dL, with glucosuria and ketonuria. EDKA was diagnosed and he was managed in the intensive care unit with fluids, electrolytes, and broad-spectrum antibiotics. His condition began to improve after 48 hours. He was found to have colonic splenic flexure cancer on further investigation, for which he underwent a left hemicolectomy. Dapagliflozin was stopped, and basal-bolus insulin was prescribed. On both occasions, the authors stated that there is no clear evidence to indicate that SGLT2i use in patients with cancer increased the risk of EDKA. They recommended that further studies be conducted to elucidate if malignancy increases the risk of EDKA.

## Conclusions

SGLT2i are now widely used to control hyperglycemia in diabetic patients. These medications are gaining popularity in diabetic management, mainly due to their proven CV and renal benefits in recent studies. The risk factors for EDKA in patients taking SGLT2i are diverse, and many preexisting or acute conditions can act as precipitants placing patients at risk for EDKA. Practitioners should be vigilant in diagnosing EDKA by thoughtful patient assessment for acidosis, measuring urine ketones even in normoglycemia. If EDKA is suspected, oral antidiabetic drugs, especially SGLT2i, should be discontinued and prompt treatment with intravenous fluids and insulin should be initiated in suspected patients. Patients also need to be educated about risk factors, triggers, signs, and symptoms and instructed to consult a physician before commencing any dietary changes. More research is needed to understand the mechanism of EDKA in the event of precipitants.
